# A Pilot Study of Electrocardiographic Features in Patients with Obesity from a Tertiary Care Centre in Southern India (Electron)

**DOI:** 10.3390/medsci10040056

**Published:** 2022-09-28

**Authors:** Aditya John Binu, Sirish Chandra Srinath, Kripa Elizabeth Cherian, John Roshan Jacob, Thomas V. Paul, Nitin Kapoor

**Affiliations:** Christian Medical College & Hospital, Vellore 632004, India

**Keywords:** obesity, ECG, cardiovascular disease

## Abstract

**Background:** Obesity is associated with increased all-cause mortality and cardiovascular disease (CVD). An electrocardiogram (ECG) may be used to screen for subtle signs of CVD or altered cardiac morphology in the obese. **Methodology:** This observational cross-sectional analysed ECG changes in patients with obesity at a tertiary care centre in southern India. **Results:** One hundred and fifty adult patients with a mean (SD) BMI of 39.9 (6.7) kg/m^2^ were recruited in the study after excluding those with comorbidities (diabetes mellitus, systemic hypertension) or on chronic medications (ACE inhibitors). The cohort showed a female predominance (69.3%), with a mean (SD) age of 45.4 (11.2) years. Most patients exhibited a sinus rhythm (78%), with one patient showing features of first-degree conduction block. Sinus tachycardia was seen in 32 (21.3%) patients. We observed left and right ventricular hypertrophy in five (3.3%) and three (2%) patients, respectively. Observed ECG patterns included a prolonged QTc in 16 (10.7%) patients, inverted T-waves (mostly in the inferior leads) in 39 (26%) patients and ST-segment depression (predominantly in the lateral leads) in 14 (9.3%) patients. A greater prevalence was noted for morbid obesity. No deaths were reported in our cohort. **Conclusions:** The predominant ECG variations in this cohort included tachycardia, atrial enlargement, ventricular hypertrophy, conduction defects, LAD, features of ischemia or old infarction and repolarization abnormalities, with a greater prevalence in morbid obesity. Further studies are needed to assess the impact of weight reducing measures on reversibility of these changes and determine the association with outcomes in obese patients.

## 1. Introduction

The epidemic of obesity has seen no respite despite a significant body of scientific literature dedicated to the evaluation of its aetiology, management and complications. Obesity in adult males and females is associated with a significant reduction in life expectancy [1]. The body mass index (BMI) is a simple index derived by dividing the subject’s weight (in kg) by the square of his or her height (in m^2^), and it is commonly used to classify underweight, overweight and obesity in adults. Obesity is defined as a BMI of >30 kg/m^2^, whereas severe or class III obesity is defined as a BMI >40 kg/m^2^ (≥35 kg/m^2^ if comorbidities are present) [2]. A greater BMI is associated with increased rate of death from all causes and cardiovascular disease (CVD), with progressively greater mortality as BMI increases to more than 25 kg/m^2^ [3].

“Metabolically healthy” obesity refers to individuals who do not suffer from cardio-metabolic abnormalities secondary to adiposity (hypertension, hypertriglyceridemia, low high-density lipoprotein [HDL] cholesterol, abnormal C-reactive protein (CRP), impaired fasting glucose and/or evidence of insulin resistance). These individuals have also been found to have a significantly increased risk of mortality and cardiovascular risk compared to metabolically healthy normal-weight individuals over 10 years of follow-up [3]. The American Heart Association (AHA) has identified obesity as an independent risk factor for coronary heart disease (CHD) [3]. A meta-analysis of 21 studies that studied the impact of body weight on CHD revealed a 29 percent increase in CHD for a corresponding five-unit increase in BMI [4]. The risk of CHD in obese and overweight persons is compounded by the coexistence of other risk factors such as systemic hypertension, dyslipidaemia, and diabetes. The risk attributable to obesity alone and the mechanisms by which obesity promotes coronary atherogenesis remain uncertain.

An electrocardiogram (ECG) may be used as a screening tool to detect subtle signs of cardiovascular dysfunction or altered cardiac morphology. ECG abnormalities in obesity described in the literature include an axial shift of the P-wave, QRS complex and T-wave to the left, a decrease in QRS complex voltages, features of left ventricular hypertrophy (LVH), changes in P-wave morphology, flattening of T-waves in the inferior and lateral leads, and a prolonged QT interval duration [5,6]. Many of these alterations are reversible with weight-reducing lifestyle modifications or following bariatric surgery. In this study, we aimed to analyse the ECG variations and abnormalities in patients with obesity under evaluation in a tertiary care centre in southern India.

## 2. Materials and Methods

This cross-sectional, observational study was conducted in a teaching hospital in southern India, in a 2858-bed multi-speciality medical institution with an estimated 10,500 patients seen in the out-patient department daily. It was conducted among patients presenting to the Bariatric Medicine Clinic of our institution. The study was conducted over a period of one year from 1 May 2019 to 30 April 2020. Patients were included if they were aged 18 years or above, with a BMI ≥30 kg/m^2^ and if the baseline ECG details were available in the medical records. Patients were excluded if ECG details were unavailable, if they were known to suffer from comorbidities (systemic hypertension, diabetes mellitus, valvular heart disease or coronary artery disease), and if they were undergoing treatment with chronic medications such as diuretics, digoxin, angiotensin-converting enzyme inhibitors (ACE-I) or angiotensin receptor blockers (ARB).

ECG changes (Table 1) were studied and tabulated in a proforma, which was designed for the purpose of analysing multiple ECG variations. Patient details were obtained from the Computerised Hospital Information Processing System (CHIPS) and analysed by a trained physician. The study was approved by the IRB of Christian Medical College, Vellore, India.

### Statistical Analyses

Categorical variables are presented as frequencies and percentages, while continuous variables are presented as mean and standard deviations after assessment for normality using the Shapiro–Wilk test. The chi-square test or Fisher’s exact test was used for comparison of nominal variables, and the two-sample *t*-test or the Mann–Whitney U test was used to compare continuous variables. A *p* value < 0.05 was considered as statistically significant. All components of the statistical analysis were performed using IBM SPSS Statistics Version 23 (IBM, Armonk, NY, USA).

## 3. Results

A total of 150 patients were included in our study after stringent application of the inclusion and exclusion criteria. As noted in Table 2, the study population was predominantly female (69.3%) with a mean (SD) age of 45.4 (11.2) years. The mean (SD) BMI of the study cohort was 39.9 (6.7) kg/m^2^. Seventy-seven percent (*n* = 116) were morbidly obese (a BMI of >35 kg/m^2^). Most patients (78%) were found to be in normal sinus rhythm (Table 3) with a mean heart rate (SD) of 89 (12) beats/minute, while 32 (21.3%) patients had sinus tachycardia. The mean PR (SD) interval was determined to be 146 (19.8) milliseconds (ms). Two patients were detected to have arrhythmias; one was found to have a first-degree atrioventricular conduction block (AV block) while the other individual had premature ventricular beats.

QRS complexes were normal in morphology and duration in the majority of patients; the mean QRS duration (SD) was 88.8 (9.6) ms. Five (3.3%) patients were found to have ECG features suggestive of a bundle branch block (BBB) with one (0.7%) patient having a left-sided (LBBB) and four (2.7%) patients having a right-sided (RBBB) block. Most patients were found to have a normal cardiac axis on the ECG. Four (2.7%) patients were found to have a left axis deviation (LAD). We observed left ventricular and right ventricular hypertrophy (LVH and RVH) in five (3.3%) and three (2%) patients, respectively; one patient was found to have ECG features of both LVH and RVH.

In the study cohort, seven (4.7%) patients and 2 (1.3%) patients had features of left atrial enlargement (LAE) and right atrial enlargement (RAE), respectively. The corrected QT (QTc) interval was assessed in all patients; the mean QTc (SD) was found to be 432.3 (22.9) ms. The QTc was found to be prolonged in 16 (10.7%) patients.

ST-segment depression was seen in 14 (9.3%) patients, with the majority (6 out of 14 (42.9%)) being in the lateral leads (I, aVL, V_5_, V_6_). Inverted T-waves were seen in 39 (26%) patients, with the majority (20 of 39 (51.3%)) occurring in the inferior leads (II, III, aVF). Ten percent of the patients were found to have Q-waves on the ECG, of whom four (2.7%) had features suggestive of pathological Q-waveforms. Poor R-wave progression was seen in 17 (11.3%) patients.

ECG features were compared between patients (Table 3) with morbid obesity (MO) and non-morbid obesity (NMO) using a cut-off of BMI >35 kg/m^2^, as defined in previous studies [7]. There was a greater frequency of tachycardia, LAE or RAE, LVH or RVH, LAD, features of ischemia/infarction (Q-waves, inverted T-waves) and repolarization abnormalities in the cohort with morbid obesity, but no clinically significant statistical differences were noted between both cohorts.

No mortality was reported in our cohort at the time of analysis.

## 4. Discussion

In this observational study, we found an increased frequency of tachycardia, atrial enlargement, ventricular hypertrophy, conduction defects (LBBB/RBBB), LAD, features of ischemia or old infarction (pathological Q-waves, poor R-wave progression, ST-segment depression and inverted T-waves) and repolarization abnormalities (prolonged QTc interval) in patients with morbid obesity.

The prevalence of obesity has been steadily increasing, and if post-2000 trends were to continue, the global prevalence of obesity is expected to reach 18% in men and surpass 21% in women by 2025 [8]. Obesity is considered to be an independent risk factor for CVD [9], and is known to cause morphological changes in the heart with a resultant alteration in the normal ECG pattern [10,11]. Morphological changes induced by obesity, such as cardiac displacement due to diaphragmatic elevation, cardiac hypertrophy secondary to increased cardiac workload, adipose tissue accumulation in subcutaneous tissue underlying the electrodes, and changes secondary to disorders such as sleep apnoea, are speculated to cause ECG changes in patients [5,6,9]. Studies have also demonstrated an increased risk of sudden cardiac death with increasing BMI and waist-to-hip ratio [12].

The classical ECG pattern in the obese is characterised by low-voltage, left axis deviation (LAD), Q_III_ and negative T_III_ [5]. As noted in our analysis, the predominant distribution of inverted T-waves was in the inferior cardiac leads. However, ST segment depression was predominantly noted in the lateral chest leads. Q-waves were noted in lead III but were not found to have any features suggestive of pathological waveforms.

Despite the increased frequency of ECG abnormalities in the MO subgroup, we could not determine any statistically significant differences in these variations compared to the NMO group. As our cohort did not suffer from any underlying comorbidities (hypertension, diabetes mellitus), it may have resulted in a decreased frequency of these ECG signs in these patients. As patients with MO have a greater probability of acquiring these complications later in life, the frequency of these variations may increase further with time.

LV hypertrophy is underdiagnosed by ECG in morbidly obese subjects, which has been attributed to excessive subcutaneous adipose accumulation in the precordium, thereby reducing the QRS voltage signal generated by the left ventricle [13]. The American Heart Association (AHA) has recommended that Sokolow–Lyon voltage criteria be replaced by the Cornell voltage criteria, as they may be less influenced by the presence of obesity [9]. LVH was noted in 3.3% of the patients in our study population.

Studies have shown that an increasing relative body mass and adiposity are associated with prolonged ventricular repolarization and significant lengthening of the QTc interval [14,15]. This has been hypothesised to be secondary to changes in cardiac autonomic balance, which includes reduced vagal tone with relatively unchanged sympathetic outflow in obese individuals [16,17].

Reasons for the LAD in morbidly obese subjects include horizontal displacement of the heart as a consequence of abdominal adiposity and the presence of LV hypertrophy [13]. Previous studies have shown a tendency toward increasing PR interval and QRS duration with increasing obesity, which were independent of age, sex and blood pressure [18,19]. BMI has also been demonstrated to be a predictor of the presence of fragmented QRS complexes on ECG, independent of underlying cardiovascular status [20]. Neither of these findings were noted amongst our study subjects.

Sudden death during dieting or post-bariatric surgery has been reported, and it is associated with delayed cardiac repolarization and prolonged corrected QT interval (QTc) [14]. Weight loss secondary to bariatric surgery brings about a proportional decrease in resting oxygen consumption and cardiac output to the magnitude of weight loss [21]. Weight reduction in obese subjects reduces the indices of sympathetic activity and decreases plasma renin activity and aldosterone levels, which have a causative role in LVH [22]. Pharmacological management of obesity is associated with a significant side-effect profile that may influence the cardiovascular system. The use of agents such as fenfluramine and dexfenfluramine was found to be associated with the risk of cardiac-valve disorders and primary pulmonary hypertension [23,24]. Sibutramine has been shown to be associated with hypertension and tachycardia [25]. Furthermore, CVOT trials are now mandatory for any anti-obesity medications before approval.

To the best of our knowledge, this is the first study assessing ECG patterns in obese patients from India. This study is limited by its cross-sectional design, small sample size and lack of a non-obese control group. Based on this study, we recommend a further study with a larger number of patients and a non-obese control. Our cohort of patients with both MO and NMO remain under follow-up in a dedicated bariatric medicine clinic to detect any resolution in these ECG variations secondary to weight reduction and lifestyle modification.

## 5. Conclusions

In our study, we analysed ECG patterns in patients diagnosed with morbid obesity under evaluation in a Bariatric medicine clinic. The predominant changes seen in this cohort included tachycardia, atrial enlargement, ventricular hypertrophy, conduction defects, LAD, features of ischemia or old infarction and repolarization abnormalities, with a greater prevalence in morbid obesity. No deaths were reported in the cohort. Further studies are needed to assess the impact of weight reducing measures on the reversibility of these changes and determine the association with outcomes in obese patients.

## Figures and Tables

**Table 1:** ECG details obtained from medical records.

S. No.	ECG Features
1.	Heart rate (Tachycardia/Bradycardia)
2.	P-wave morphology/duration
3.	PR interval
4.	Q-waves (Pathological/Normal)
5.	QRS wave morphology/duration
6.	Corrected QT interval (QT_c_)
7.	Axis deviation (Left/Right/Normal)
8.	Arrhythmias
9.	ST segment abnormalities
10.	T-wave abnormalities
11.	Bundle Branch Blocks (Left/Right)
12.	Ventricular Hypertrophy (Left/Right)
13.	Poor R-wave progression

**Table 2:** Baseline characteristics.

Variable	N (%)	
**Gender**		
Male	46 (30.7)	
Female	104 (69.3)	
	Mean (SD)	Range (Min–Max)
Age (years)	45.4 (11.2)	25–69
Height (cm)	159.8 (9.7)	145–189
Weight (kg)	101.7 (20.3)	51–146
Body mass index (kg/m^2^)	39.9 (6.7)	25–53

**Table 3:** Summary of ECG features: comparison of patients with morbid obesity (MO) and non-morbid obesity (NMO)].

ECG Features	Patients with MO [N (%)]	Patients with NMO [N (%)]	*p*-Value
**Heart rate**	88 (75.9) 27 (23.3) 1 (0.8)	29 (85.3) 5 (14.7) 0 (0.0)	0.47
Normal rate Sinus tachycardia Sinus bradycardia			
**P-wave morphology**	109 (94.0) 5 (4.3) 2 (1.7)	32 (94.1) 2 (5.9) 0 (0.0)	0.70
Normal Left atrial enlargement Right atrial enlargement			
**PR interval**	105 (90.5) 1 (0.9) 10 (8.6)	32 (94.1) 0 (0.0) 2 (5.9)	0.75
Normal Prolonged Shortened			
**Q-waves**	112 (96.6) 4 (3.4)	34 (100.0) 0 (0.0)	0.52
Normal Pathological			
**Corrected QT interval (QT_c_)**	102 (87.9) 0 (0.0) 14 (12.1)	32 (94.1) 0 (0.0) 2 (5.9)	0.53
Normal Shortened Prolonged			
**Axis**	112 (96.6) 4 (3.4) 0 (0.0)	34 (100.0) 0 (0.0) 0 (0.0)	0.58
Normal Left sided deviation Right sided deviation			
**Arrhythmias**	2 (1.7)	0 (0.0)	0.89
**ST segment**	105 (90.5) 11 (9.5)	31 (91.2) 3 (8.8)	0.90
Normal Depression			
**T-wave abnormalities**	29 (25.0)	10 (29.4)	0.61
**Bundle Branch Blocks**	1 (0.9) 4 (3.5)	0 (0.0) 0 (0.0)	0.47
Left bundle branch Right bundle branch			
**Ventricular Hypertrophy**	5 (4.3) 3 (2.6) 1 (0.9)	0 (0.0) 0 (0.0) 0 (0.0)	0.42
Left Right Biventricular hypertrophy			
**Poor R-wave progression**	15 (12.9)	2 (5.9)	0.36
**Average interval length [Mean (SD)]**			
**PR Interval**	146.4 (20.2)	144.8 (18.6)	0.67
**QRS Duration**	89.3 (10.1)	87.1 (11.4)	0.13
**QTc interval**	433.6 (23.8)	427.71 (19.1)	0.17

## Data Availability

The data presented in this study are available on request from the corresponding author. The data are not publicly available due to sensitive nature of data of patients from the clinic.
